# Physicochemical Properties, Drug Release and In Situ Depot-Forming Behaviors of Alginate Hydrogel Containing Poorly Water-Soluble Aripiprazole

**DOI:** 10.3390/gels10120781

**Published:** 2024-11-29

**Authors:** Hy D. Nguyen, Munsik Jang, Hai V. Ngo, Myung-Chul Gil, Gang Jin, Jing-Hao Cui, Qing-Ri Cao, Beom-Jin Lee

**Affiliations:** 1 Department of Pharmacy, College of Pharmacy, Ajou University, Suwon 16499, Republic of Korea; hynguyendinh@ajou.ac.kr (H.D.N.); anstlr93@naver.com (M.J.); haingo25895@gmail.com (H.V.N.); kilmch88@naver.com (M.-C.G.); 2 School of Chemical and Pharmaceutical Engineering, Jilin Institute of Chemical Technology, Jilin 132022, China; jingang181217451@163.com; 3 College of Pharmaceutical Sciences, Soochow University, Suzhou 215123, China; jhcui@suda.edu.cn (J.-H.C.); qrcao@suda.edu.cn (Q.-R.C.); 4 Institute of Pharmaceutical Science and Technology, Ajou University, Suwon 16499, Republic of Korea

**Keywords:** poorly water-soluble aripiprazole, alginate hydrogel, in situ depot-forming behaviors, ratio of pyridoxal phosphate and glucono-delta-lactone, controlled gelation time, physicochemical properties, drug release rate

## Abstract

The objective of this study was to investigate the physicochemical properties, drug release and in situ depot-forming behavior of alginate hydrogel containing poorly water-soluble aripiprazole (ARP) for achieving free-flowing injectability, clinically accessible gelation time and sustained drug release. The balanced ratio of pyridoxal phosphate (PLP) and glucono-delta-lactone (GDL) was crucial to modulate gelation time of the alginate solution in the presence of calcium carbonate. Our results demonstrated that the sol state alginate hydrogel before gelation was free-flowing, stable and readily injectable using a small 23 G needle. In addition, the ratio (*w*/*w*) of PLP and GDL altered the gelation time, which was longer as the PLP content increased but shorter as the GDL content increased. The alginate hydrogel with a ratio of PLP to GDL of 15:9 had the optimal physicochemical properties in terms of a clinically acceptable gelation time (9.1 min), in situ-depot formation with muscle-mimicking stiffness (3.55 kPa) and sustained release over a two-week period. The alginate hydrogel, which is tunable by varying the ratio of PLP and GDL, could provide a controllable pharmaceutical preparation to meet the need for long-acting performance of antipsychotic drugs like ARP.

## 1. Introduction

Aripiprazole (ARP) is a second-generation antipsychotic medication widely used to manage and treat psychotic disorders such as schizophrenia, mania associated with bipolar I disorder, autism spectrum disorder and Tourette syndrome [1]. ARP, classified as Biopharmaceutics Classification System (BCS) Class II, has pH-dependent and poor solubility above pH 5 due to its weakly acidic nature (pKa = 7.6) [2]. Various ARP dosage forms are currently available, such as oral tablets, oral tablet embedded with ingestible event marker sensors (Abilify MyCite) and oral disintegrating tablets [2,3]. However, patients with schizophrenia often experience emotional and cognitive dysfunctions and exhibit negative attitudes toward conventional oral dosage forms; thus, the frequent self-administration leads to poor patient adherence, with non-adherence rates reported to be as high as 72% [4]. Therefore, there is a strong need to develop long-acting injectable (LAI) ARP that provides a sustained drug release to improve patient adherence according to the severity of disease in schizophrenia treatment [1,3].

Several pharmaceutical approaches have been developed to design LAIs for antipsychotic drugs, including poly (lactic-co-glycolic acid) (PLGA)-based microspheres, oil-based solutions and aqueous-based nanosuspensions containing drug or lipophilic prodrug obtained by esterification with long-chain aliphatic fatty acids such as decanoic acid or palmitic acid [5,6]. However, each approach has its limitations. Oil-based injectables in sesame or coconut oil can cause unwanted pain at the site of injection and patient discomfort [7]. Nano/microsphere-based formulations require complicated manufacturing processes with special machines for reducing particle size, such as wet grinding, jet pulverization and high-pressure homogenization, in addition to the use of harmful organic solvents [8,9]. In the case of PLGA-based microspheres, the drug release kinetics are difficult to predict. Many PLGA-based formulations usually occur with an initial burst release due to the rapid diffusion of the drug loosely associated with the particles’ surface or through the pores or cracks of microspheres, which can shorten the overall duration and cause adverse effects [10]. In contrast, the initial degradation of the microspheres of a few formulations, such as Risperdal Constar, is too slow to release the drug after injection, requiring inconvenient oral administration at the initial stage to maintain the plasma concentration of the drug above the minimum effective concentration [5]. An existing LAI suspension of ARP (Abilify Maintena^®^) is available, but, similar to other nano/microparticle formulations, challenges remain regarding the patient discomfort caused by invasive large needle sizes (21–23 G) injection, the initial burst release, the risk of physical instability and the complicated manufacturing process [11,12].

To circumvent these limitations, in situ forming gel (ISFG) systems can be an alternative to conventional LAI methods owing to their ease of administration, controllable release profiles and simple preparation process. ISFGs can improve the efficiency of delivery due to the ease of administration via thin-gauge needles, such as 29 G needles. Unlike nano/microparticle formulations, the simple design of hydrogels avoids the potential physical instability and ununiform drug distribution. In addition, ISFG utilizes biocompatible and biodegradable polymers, avoiding local irritation and toxicity [13]. Different water-swellable polymers, such as gelatin, alginate, chitosan, gellan gum or synthetic polymers can be used in ISFG systems. A polymer-based ISFG can be injected in a liquid state but converted into a gel or solid depot in the body via physical, chemical, pH and thermally induced liquid-to-solid phase inversion. The depot then slowly releases the drug over a prolonged period, providing sustained drug delivery [13]. For example, Oncogel contains a PLGA-PEG-PLGA polymer that presents in solution form at room temperature but undergoes a phase inversion to form a biodegradable water-insoluble gel depot upon being injected into the body. For 6 weeks, Oncogel continuously delivers paclitaxel to the tumor along with the surrounding tissue [14].

Alginate, a natural polysaccharide obtained from brown algae, has demonstrated its pharmaceutical advantages, including its good physical properties such as excellent biocompatibility, non-toxicity and cost-effectiveness [15], and its potential as a drug delivery biomaterial for zwitterionic polymer and wound dressing [16,17]. Its guluronate blocks can attach to calcium cations to form an egg-box structure, along with a three-dimensional hydrogel [18]. Recently, alginate hydrogel was reported as an ISFG for medical and pharmaceutical applications in long-acting drug delivery [13]. Without organic solvent usage, an ISFG system of alginate hydrogel was injected in a free-flowing liquid state using a smaller-gauge needle but converted into a rigid gel or solid depot at the site of injection through combination with calcium cations [19,20]. However, the rapid cross-linking reaction between alginate and calcium cations can result in poor injectability and an inconsistent hydrogel, while the long gelation time might lead to a large initial burst release [21]. Therefore, modulation of the gelation time of alginate-based ISFGs from 3 to 10 min is very crucial for clinically accessible parenteral administration [20]. An appropriate combination of glucono-delta-lactone (GDL) and pyridoxal phosphate (PLP) has been demonstrated for controlling gelation behaviors [20,22,23].

**Scheme 1:**
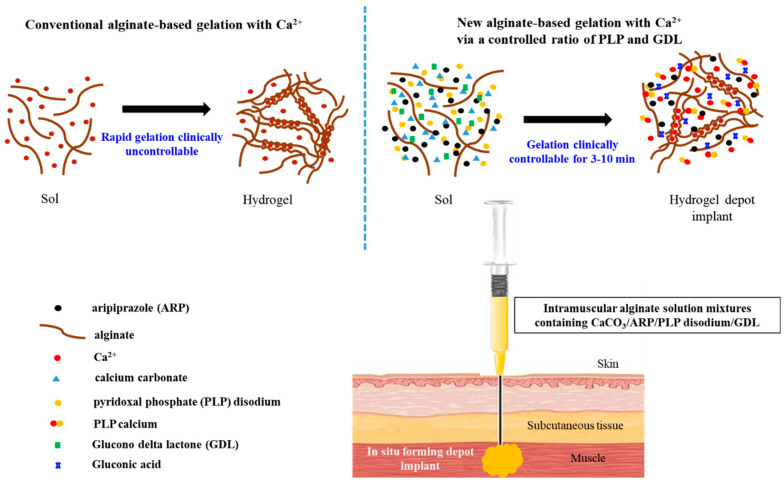
Schematics of a long-acting injectable alginate hydrogel and the mechanism of gelation time control using glucono-delta-lactone (GDL) and pyridoxal phosphate (PLP).

In this study, we aimed to prepare a simple ARP-loaded alginate ISFG by modulating the ratios of GDL and PLP to achieve patient-friendly options such as free-flowing injectability, a clinically accessible gelation time and sustained drug release for long-term therapy. The physicochemical properties of the alginate hydrogel, such as its injectability, swelling ratio, in vitro degradation rate, injection site stiffness, change in viscosity during storage and drug release profiles, were characterized. The surface morphology of the alginate hydrogel was evaluated using scanning electron microscopy (SEM). The in situ-depot-forming properties of the alginate hydrogel were investigated by injecting the sol state into the muscular tissue of pig necks. The in vitro drug release of the alginate hydrogel was carried out with a dissolution tester using USP apparatus II or a shaking bath method, compared with a commercial ARP product (Abilify Maintena). Scheme 1 illustrates the schematic of a long-acting injectable alginate hydrogel and the mechanism of controlling gelation time using GDL and PLP.

## 2. Results and Discussion

### 2.1. Formation of the Alginate Hydrogel

The preparation of the alginate hydrogel was similar to that in our previous study, with some modifications [20]. Briefly, alginate hydrogel was prepared by adding GDL (5 mL) to a pregel solution that contained sodium alginate (0.6 g), ARP (0.84 g), calcium carbonate (CaCO_3_, 0.6 g) and PLP disodium (25 mL), followed by homogenous mixing. The ARP dose was based on a daily dose of 10 mg/day. For the long-acting duration of 14 days, a total dose of 140 mg/5 mL or 0.84 g/30 mL was prepared. If calcium chloride is used as the calcium source for the gelation of an alginate-based hydrogel, the calcium cations will react quickly with alginate to form a non-homogeneous hydrogel [24,25]. In contrast, due to the poor solubility of CaCO_3_ salt, the concentration of calcium cations is very low and was not enough to trigger a sol-to-gel transition in the pregel solution. Upon the addition of GDL, calcium cations were gradually released from CaCO_3_, corresponding to the change from a neutral to an acidic environment, and then reacted with alginate to form a stable and uniform hydrogel [19,26]. Different formulations were prepared to evaluate the effect of the PLP/GDL ratio on gelation time (Table 1).

**Figure 1:**
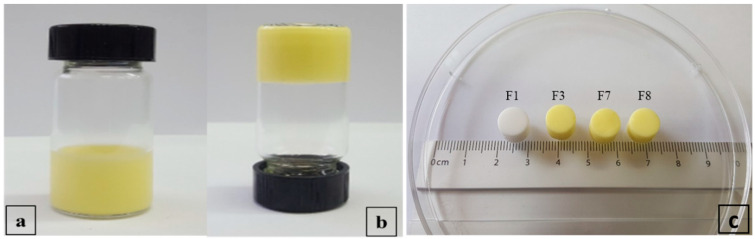
Physical appearance of alginate hydrogel in (**a**) the sol state and (**b**) the gel state, and (**c**) the hydrogel containing a fixed ARP dose of 14 mg.

Figure 1 shows the physical appearance of the alginate hydrogel in the sol state and the gel state, and the hydrogel containing a fixed dose of 14 mg ARP. The sol-to-gel transition of alginate hydrogels, in which the formed hydrogel did not flow upon the reversion of the vial, is clearly visualized (Figure 1a,b). The hydrogel must be manufactured consistently in a uniform and constant shape to characterize the physicochemical and mechanical properties and perform the in vitro dissolution test. In this study, a 48-well cell culture plate (9.75 mm in diameter, 17.50 mm in height) was used as the mold to prepare the alginate hydrogel containing the ARP dose of 14 mg. The hydrogel (0.5 mL) was poured into the cell culture plate and left for gelation, so it had a cylindrical shape with a diameter of 9.75 mm and a height of 10.0 mm (Figure 1c).

### 2.2. Effect of the PLP/GDL Ratio on Gelation Time

**Figure 2:**
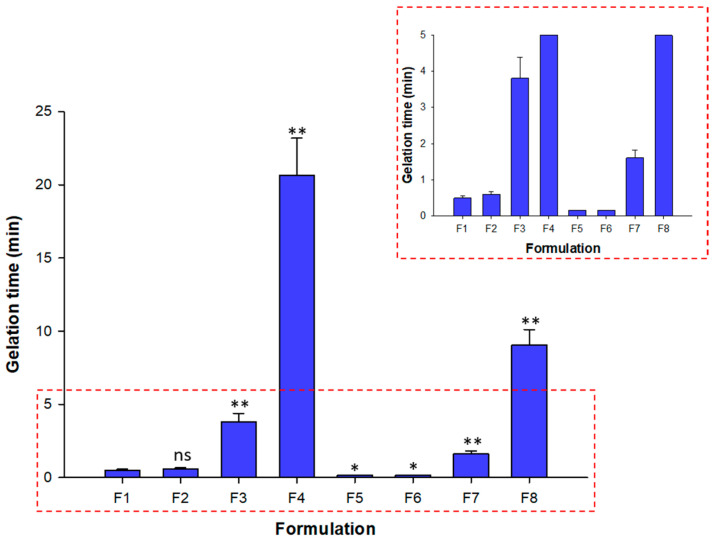
Gelation time of alginate hydrogel formulations. Data are expressed as the mean ± standard deviation (*n* = 3). The significant differences were analyzed compared with F1 by Student’s *t*-test: ** *p* < 0.01, * *p* < 0.05, and ns: no significant difference (*p* > 0.05). A detailed statistical analysis is presented in Table S1.

The in situ depot-forming behavior of alginate hydrogel was injected in a sol state, but gelation must occur in the body after a certain time to form a depot at the injection site for extended drug release. Figure 2 shows that the GDL/PLP ratio significantly affected the gelation time of the alginate hydrogel. At similar amounts of PLP, four formulations with lower amounts of GDL of 0.14 g (F1–F4) showed longer gelation time than those containing 0.18 g of GDL (F5–F8). The difference in the gelation time was due to a higher concentration of calcium cations released to react with the alginate when a higher amount of GDL was added [22]. When the GDL content remained unchanged, the time for the sol-to-gel transition tended to increase as a function of the amount of PLP. The gelation time was very short in formulations containing less than 0.26 g of PLP (F1, F2, F5 and F6) but it significantly increased for the other formulations. Furthermore, the gelation time controlled by PLP was very sensitive, being about 5.5 times higher when PLP increased from 0.28 g to 0.3 g. These results indicated the importance of PLP in controlling the gelation of alginate hydrogel, which can form PLP calcium, thereby decreasing the concentration of free calcium cations [20].

The time taken to change from sol to gel is crucial in clinical situations where the injections are actually administered to patients and to achieve a stable, sustained release of the drug [20]. If the gelation time is less than one minute, the too-short time required for injection can be stressful for both clinicians and patients. Conversely, if the gelation time is longer than 20 min, the depot may not form at the injection site, and the gel may diffuse into the surrounding fluid or easily decompose, resulting in an initial burst release or unsuccessful long-acting performance. Therefore, it is essential to adjust the gelation time appropriately, from 3 to 10 min. Formulations F3, F7 and F8 demonstrated optimal gelation times of approximately 3.8, 1.6 and 9.1 min, respectively. Therefore, they were selected for further investigation, with F1 serving as the control.

### 2.3. Characterization of Alginate Hydrogels

#### 2.3.1. Scanning Electron Microscopy (SEM)

**Figure 3:**
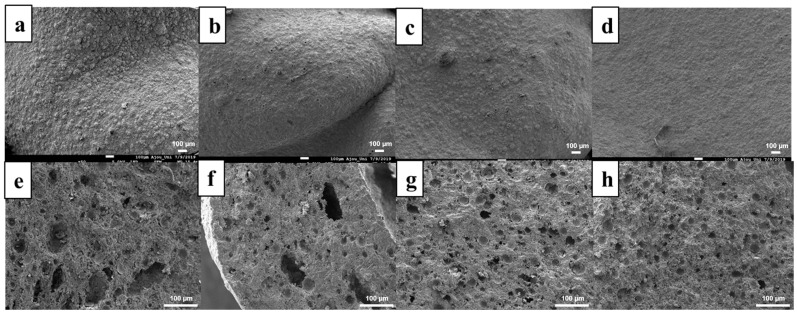
SEM images of the surface morphologies (×50) of dried alginate hydrogels after the gelation process: (**a**) F1, (**b**) F3, (**c**) F7 and (**d**) F8. Cross-sectional morphologies (×200) of (**e**) F1, (**f**) F3, (**g**) F7 and (**h**) F8.

The surface and cross-sectional morphology of the hydrogel formulations (F1, F3, F7 and F8) were observed using FE-SEM (Figure 3). F1, devoid of PLP, demonstrated the roughest surface due to the rapid gelation. The irregular structure of F1, with a rough surface and heterogenic pore size, can make the hydrogel more susceptible to swelling and degradation due to rapid water uptake, which could accelerate drug release [27]. All hydrogel formulations presented surface pore formation due to air being released during the drying process. Formulation F8, containing the highest PLP content, had the smoothest surface compared with Formulas F3 and F7. The cross-sectional morphologies of the alginate hydrogels were observed in three-dimensional porous structures to investigate the microstructure of the hydrogels. The average pore size tended to decrease when PLP was increased, indicating a higher hydrogel density for F7 and F8 than for F3 and F1. The heterogenic and large pore sizes (approximately 100 µm) seen in formulations F1 and F3 (Figure 3e,f) might lead to initial burst releases due to rapid drug diffusion through the porous regions. In contrast, the denser and more compact hydrogels with smoother surfaces for F7 and F8 (Figure 3g,h) likely contributed to sustained drug release profiles [28]. Notably, F8 exhibited the smoothest surface and uniform pore formation of around 5–10 µm.

#### 2.3.2. Swelling Test

**Figure 4:**
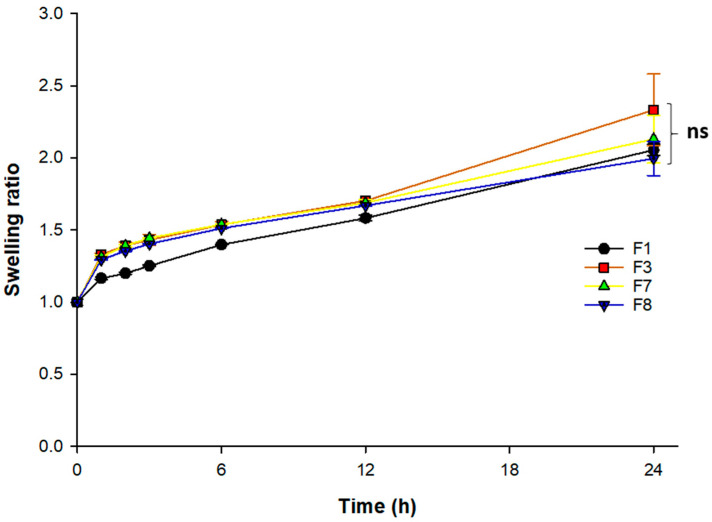
Swelling ratio of alginate hydrogel formulations. Data are expressed as the mean ± standard deviation (*n* = 3). The significant differences at 24 h were analyzed by Student’s *t*-test. ns: no significant difference (*p* > 0.05).

The swelling effect of the hydrogel scaffold is important to avoid pain at the injection site after intramuscular injection and for stable sustained release of the drug. The alginate hydrogel has a porous three-dimensional structure, which absorbs the surrounding water and swells. Following intramuscular injection, the hydrogel is formed on the muscle’s surface or the outside. The excessive swelling of hydrogel formed on the outside of the muscle by absorbing extracellular fluid can cause pain and irritation. Therefore, it is imperative to control the swelling effect to a degree that does not cause pain and discomfort. As shown in Figure 4, the swelling of alginate hydrogels rapidly reached the ratio of 1–2 and was nearly unchanged over 24 h of incubation at 37 °C in a PBS solution (pH = 7.4). The swelling ratios demonstrated no significant differences among the four formulations (Table S2). With a proper swelling rate, the shape and mechanical stability of the hydrogel could be maintained to minimize pain and irritation [29]. On the other hand, the structure of the hydrogel scaffold is one of the major factors affecting the drug release behavior, especially the initial burst release. The matrix structure formed by swelling contributed to the sustained release of the drug by inhibiting the escape of drug molecules through simple diffusion [30].

#### 2.3.3. Degradation Test

**Figure 5:**
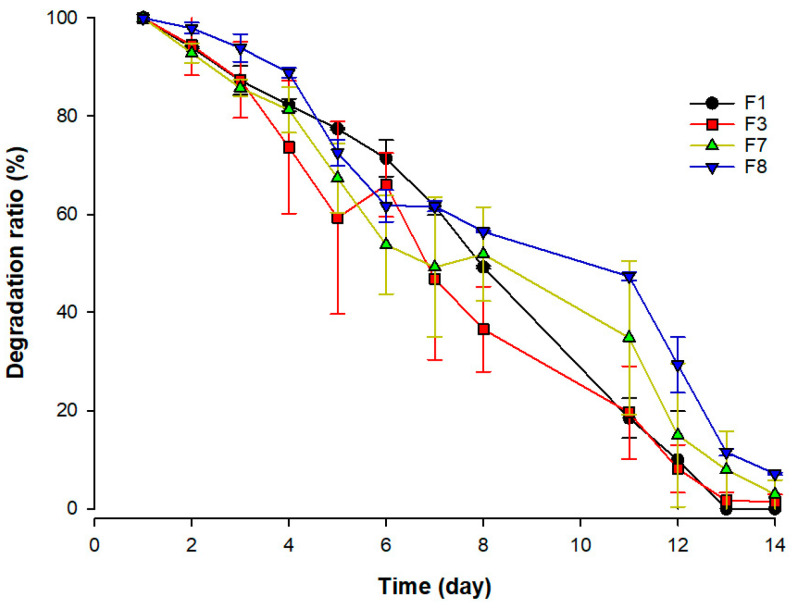
Degradation rate of alginate hydrogel formulations. Data are expressed as the mean ± standard deviation (*n* = 3). The significant differences were analyzed on Day 6 and Day 14 by Student’s *t*-test; ns: no significant difference (*p* > 0.05).

The gel degradation rate is affected by the type of polymer, the degree of cross-linking reaction and molecular interactions, as well as the elastic modulus, hydrogel pore size and water uptake. The hydrogel’s degradation rate has a direct effect on the drug release pattern [31]. For the sustained release of a drug, it should be designed to degrade as slowly as possible during the targeted period. Figure 5 shows the degradation rate of alginate hydrogels. There was no significant difference in the degradation rate among the hydrogel formulations until Day 6 and was at approximately 40%, but differences increased from Day 7 post-incubation (Table S3). While the decomposition of F1 and F3 was accelerated and they had completely degraded on Day 13, the decomposition of F7 and F8 was relatively retarded. The swelling ratios among the hydrogel formulations were similar; hence, the difference in the degradation rates was not due to hydrolysis by water uptake but likely resulted from the GDL/PLP composition. Compared with F3, Formulas F7 and F8 contained a higher amount of GDL, the gelation triggering agent, so the degree of the cross-linking reaction increased, resulting in a slower degradation rate [28,32]. Interestingly, the degradation of F8 was relatively slower than that of F7. This could be attributed to the increased molecular interactions between the alginate and PLP, which was present at a higher concentration in F8. That is, PLP containing phosphate ions could have a strong salting-out effect, resulting in enhanced hydrophobic interactions with the alginate molecules, allowing the formation of a compact and stable internal structure [33]. Additionally, PLP contributed to pore reduction by controlling the release of calcium cations, resulting in a more uniform dispersion of calcium throughout the hydrogel before the cross-linking occurred [23,34].

#### 2.3.4. Compressive Test

Hydrogels’ stiffness should be adjusted, considering the two factors of patient comfort and stable depot formation after intramuscular injection. Notably, hydrogels’ stiffness is affected by the degree of cross-linking, the swelling effect caused by water uptake and molecular interactions. If the rigidity of the depot formed on the surface and outside of the muscle is too strong, it may induce pain and irritation by pressing on the surrounding muscles. In severe cases, it may inhibit peripheral angiogenesis and interfere with cell formation, resulting in tissue necrosis [35]. Conversely, weak stiffness prevents the formation of a stable depot at the injection site, and the formed gel is easily degraded, resulting in the burst release of the drug. Therefore, it is necessary to adjust the rigidity within an optimal range to be compatible with muscular tissues, ideally lower than the stiffness of skeletal muscle (about 9~10 kPa), considering that the injection site is ventrogluteal or the rectus femoris muscle [36].

**Figure 6:**
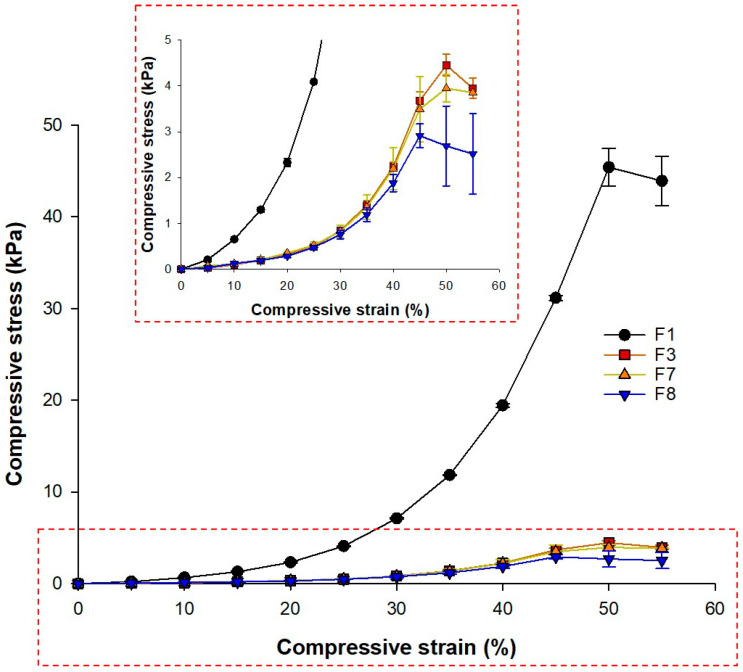
Compressive stress–strain curve of alginate hydrogel formulations. Data are expressed as the mean ± standard deviation (*n* = 3).

Figure 6 shows the compressive stress–strain curves of alginate hydrogels (F1, F3, F7 and F8). F3 and F7 presented maximal compressive stress of 4.72 kPa and 4.64 kPa at 50% compressive strain, respectively, and F8 exhibited a value of 3.55 kPa at 45% compressive strain (Table 2). These PLP-containing formulations presented properly adjusted compressive stress values, mimicking that of muscular tissues. By contrast, F1 demonstrated a maximal compressive stress of approximately 48 kPa at 50% compressive strain, which was extremely strong compared with the stiffness of skeletal muscle.

### 2.4. In Vitro Dissolution Studies

#### 2.4.1. Dissolution Test Using USP Apparatus II

**Figure 7:**
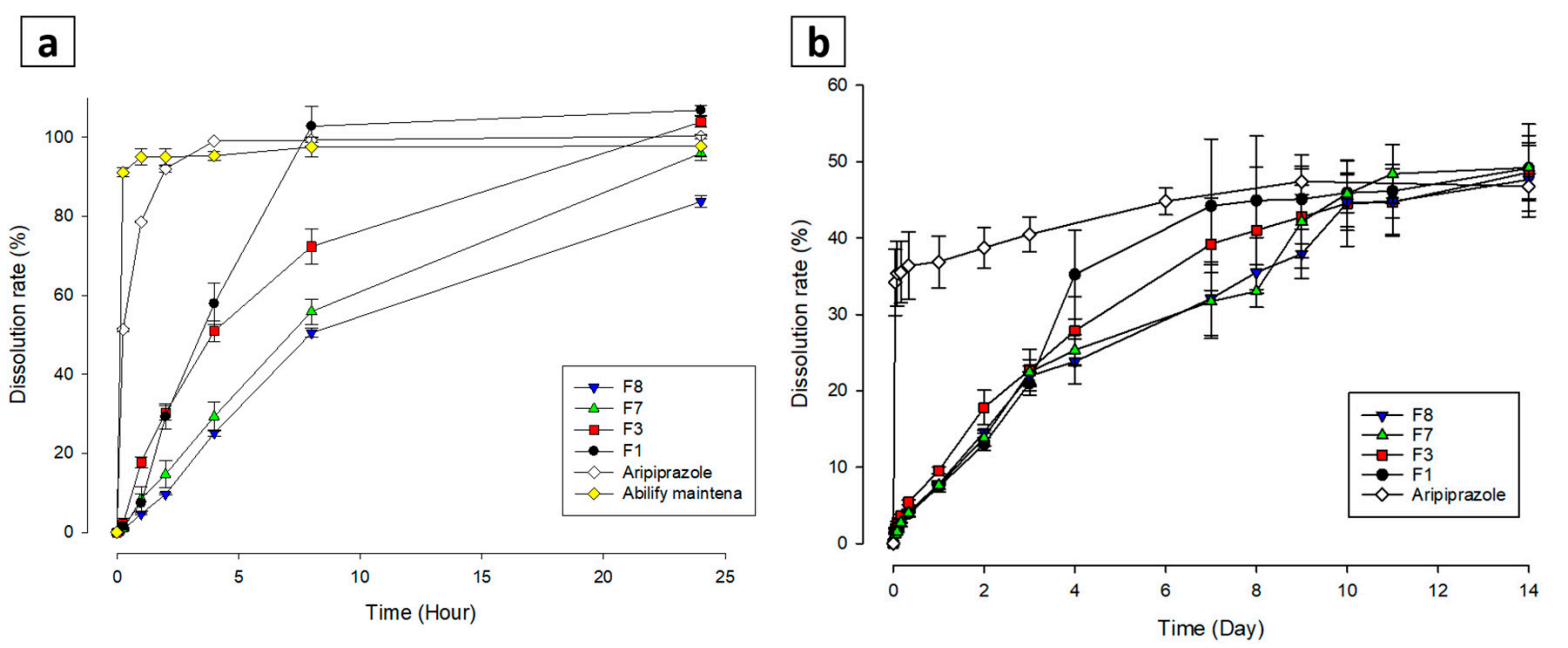
In vitro dissolution profiles of Abilify Maintena^®^, pure ARP powder and alginate hydrogels using (**a**) USP Apparatus II and (**b**) the shaking bath method. Data are expressed as the mean ± standard deviation (*n* = 3).

The in vitro dissolution test was performed using the USP paddle to confirm the dissolution rate, compared between the hydrogel formulations and the commercial product Abilify Maintena^®^. Since the sink condition was maintained at 900 mL of dissolution media containing 0.25% sodium lauryl sulfate (SLS) as a surfactant, all test formulations demonstrated a relatively fast dissolution rate of more than 80% after 24 h. The dissolution test in this condition is significant for comparing the release kinetic behavior between the test formulation and the reference product, especially at the initial stage, even though it could not completely mimic the in vivo condition at the injection site, which has a limited volume [37,38]. The hydrogel formulations exhibited a more controlled release profile compared with ARP powder and Abilify Maintena^®^, which were rapidly released within 2 h (Figure 7a). Among the four hydrogel formulations, F1 was also rapidly eluted in 8 h, while the other PLP-containing formulations had more sustained release profiles, with F8 being the formulation with the most controlled release. This result indicated that the alginate ISFG exhibited a more controlled drug release without an initial burst effect as compared with the commercial product, Abilify Maintena. Formulations F7 and F8, with higher cross-linking density or a denser structure, could delay the drug’s diffusion from the matrix gel to the dissolution media, minimizing the huge initial burst release [39].

#### 2.4.2. Dissolution Test Using the Shaking Bath Method

The in vitro dissolution test was also performed in 50 mL of a PBS solution (pH 7.4 ± 0.1) containing 0.25% SLS at 37 °C for 14 days using a shaking bath (BS-06, JEIO TECH, Republic of Korea), following the dissolution method for implant products [40]. The small volume of dissolution media was used to mimic the in vivo conditions following intramuscular injection [37,38]. Figure 7b shows the in vitro dissolution profile of the alginate hydrogels and pure ARP powder using a shaking bath (*n* = 3). It appears that the release of ARP depended on a combination of drug diffusion and the degradation of the alginate hydrogel, with diffusion playing a larger role at the beginning and corrosion of the hydrogel becoming more significant later on [41]. Similar to the dissolution test using a dissolution tester, ARP powder was rapidly released within a few hours, as there were no barriers that affected the drug’s diffusion. However, the dissolution rate reached the saturated level and increased slowly due to the low solubility of ARP in a limited volume of dissolution media [2]. By contrast, all ARP-loaded hydrogel formulations demonstrated a sustained drug release profile with similar dissolution rates until Day 3 due to the retarded diffusion of ARP from the hydrogel matrix to the surrounding media, and the differences among the formulations increased from Day 4 to Day 14, when hydrogel gradually degraded. Formulation F1 reached the maximum dissolution rate on Day 7, while the other formulations (F3, F7 and F8) showed a sustained release over 14 days. These drug release kinetics were similar to the degradation test (Figure 5), with slower degradation rates for F7 and F8 compared with F3 and F1. The explanation, as discussed earlier, was due to the increased density of alginate hydrogel in the presence of PLP. These results demonstrated that the alginate hydrogel can be used for sustained drug delivery over an extended period with a minimized burst effect, allowing a dosing frequency of once or twice per month. Overall, Formulation F8 exhibited the most controlled drug release profile without an initial burst release but with sufficient gelation time and muscle-mimicking stiffness, so it is considered as a potential formulation for LAI ARP.

### 2.5. Stability, Injectability and In Situ Depot-Forming Behavior of Alginate Hydrogels

#### 2.5.1. Stability Test of the Sol State

**Figure 8:**
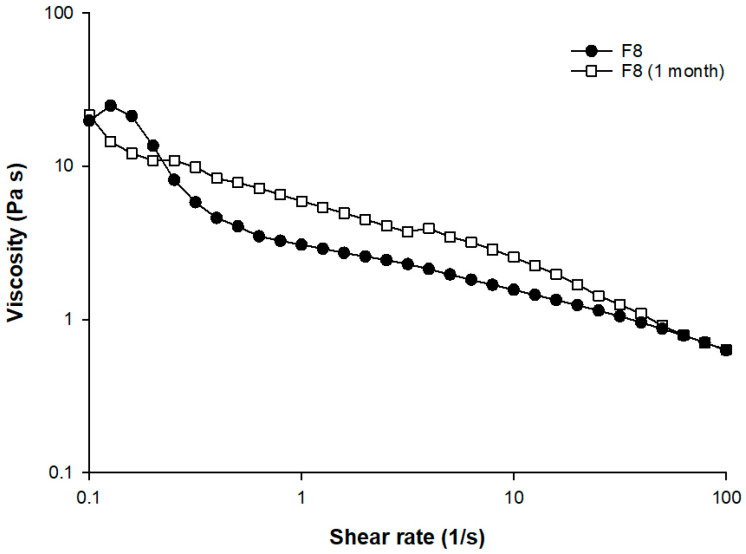
Viscosity of the pregel solution (F8) after 1 month of storing at 25 °C.

The GDL solution and the pregel solution containing the mixtures of alginate/CaCO_3_/ARP/PLP disodium must be stored separately before injection. However, even in the absence of GDL, the viscosity of the pregel solution may be increased owing to the reaction between the calcium cations released from CaCO_3_ and the alginate over a long period of storage. The stability test was performed by comparing the viscosity as a function of the shear rates from 0.1 to 100 s^−1^ of the pregel solution stored for 1 month at 25 °C. There was nearly unchanged viscosity of the pregel solution during storage (Figure 8), indicating that no cross-linking reaction occurred and that the formulation could be stored at room temperature without physical stability issues.

#### 2.5.2. Injectability and In Situ Depot-Forming Behavior of Hydrogel Implants in Pig Neck Muscle

**Figure 9:**
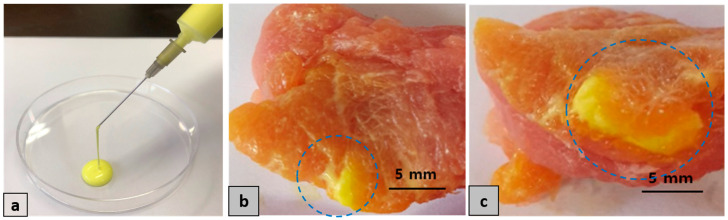
(**a**) Injectability of the sol state using a 23 G needle and (**b**,**c**) in situ depot-forming behavior of alginate hydrogel (F8) in pig muscle, showing (**b**) surface and (**c**) cross-sectional morphological images.

The sol state obtained after mixing the GDL solution with the pregel solution containing alginate/CaCO_3_/ARP/PLP disodium should be injectable with a syringe prior to gelation. If the viscosity of the sol state is too high, it may fail to flow through the syringe needle, or a large-gauge syringe needle must be used, which can cause patient discomfort. The injectability of Formulation F8 in the sol state was possible using a patient-friendly 23 G needle (Figure 9a).

Following the intramuscular injection, the hydrogels were exposed to the bloodstream and body fluids under physiological conditions. Therefore, it was crucial to verify the proper occurrence of gelation in biomimicking conditions. Figure 9b,c show images of the surface and cross-section of pig neck muscule tissue where the formulation was injected. A yellowish depot of alginate hydrogel was observed at the injection site without diffusion to external tissues, indicating the successful formation of in situ depot of alginate hydrogel, followed by a sustained release of ARP.

## 3. Conclusions

In this study, alginate hydrogel was prepared, and the optimized formulation (F8) was selected for the long-acting delivery of ARP. GDL and PLP were important for controlling the gelation time, improving the mechanical properties of intramuscular injection and delaying degradation by stabilizing the internal structure of the hydrogel. When the ratio of GDL to PLP was 15:9, an optimal gelation time of around 9 min was obtained for accessible patient administration. Furthermore, PLP prevented muscle damage and pain by adjusting the stiffness of hydrogel to slightly lower than that of muscular tissue. Notably, PLP increased the density and stability of the hydrogel depot by infiltrating into the internal structure, achieving controlled release of ARP for at least 14 days with a minimized initial burst effect under in vitro conditions. The current ISFG with clinically acceptable gelation time, free-flowing injectability and sustained release of ARP could provide a new drug delivery system to improve demand and adherence for psychoactive patients. The simplicity of the manufacturing process and scaling up of alginate ISFGs without using organic solvents can be easily adaptable for long-acting injectable formulations of other poorly water-soluble drugs by varying the ratio of PLP and GDL.

## 4. Materials and Methods

### 4.1. Materials

ARP and Abilify Maintena^®^ were kindly supplied by Dongwha Pharm Co., Ltd. (Suwon, Republic of Korea). Sodium alginate (Protanal LFR 5/60, G/M 65%) was purchased from Junsei Chemical Co., Ltd. (Tokyo, Japan). CaCO_3_ (98.0%) and sodium hydroxide (beads, 98.0%) were purchased from Samchun Chemicals (Seoul, Republic of Korea). GDL and PLP were purchased from Sigma-Aldrich (Saint Louis, MO, USA). All solvents used for the analytical methods were of high-performance liquid chromatography (HPLC) grade. Water was purified by reverse osmosis.

### 4.2. Preparation and Characterization of Alginate Hydrogels

#### 4.2.1. Preparation of Alginate Hydrogels

A pregel solution was prepared by mixing sodium alginate (0.6 g), ARP (0.84 g), CaCO_3_ (0.6 g) and a PLP disodium solution to form a uniform suspension (25 mL) using a vortex mixer. The PLP disodium solution was prepared by dissolving PLP and sodium hydroxide in 5 mL of distilled water at a ratio of 1:0.322 (*w*/*w*). Alginate hydrogel was prepared by adding the GDL solution (5 mL) to the pregel solution, followed by homogenous mixing for around 30 s. The resulting mixture (0.5 mL) was poured into a 48-well plate (9.75 mm diameter, 17.50 mm height) for solidification through sol–gel transformation.

#### 4.2.2. Measurement of the Gelation Time

Following the mixing of the GDL solution with the pregel solution, the hydrogel was formed through sol–gel transformation. The gelation time was measured by visually observing the gel’s fluidity using a vial inversion test according to the definition, that is, the time taken for the sol state to halt its fluidity for 30 s after the inversion of the test vial [42].

#### 4.2.3. Scanning Electron Microscopy (SEM)

The surface morphologies and the cross-section of the F1, F3, F7 and F8 hydrogel formulations were characterized by FE-SEM (field emission scanning electron microscopy, JSM-7900F, JEOL, Tokyo, Japan) at 5 kV. The samples (1–1.5 mg) were placed on a SuS stage using carbon tape and coated with platinum for 2 min in air.

#### 4.2.4. Swelling Test

The swelling ratio of the hydrogels was analyzed by incubation in phosphate-buffered saline (PBS) for 24 h. The diameter and height of the hydrogel were 1 cm and 0.7 cm, respectively. The hydrogels were weighed and incubated in 30 mL of the PBS solution (pH 7.4 ± 0.1) using a water bath (BS-06, JEIO TECH, Daejeon, Republic of Korea). The incubation conditions were set to 37 ± 0.5 °C and a rotation speed of 50 rpm. The hydrogels were removed from the PBS solution at 1, 2, 3, 6, 12 and 24 h. The excess water on the surfaces was removed using filter paper, and the weight was recorded. The swelling ratio (SR) was calculated according to the following equation

(1) $$\mathrm{SR}=\frac{Ws}{Wi}$$

where *W_i_* is the weight of initial hydrogel and *W_s_* is the weight of swollen hydrogel after incubation at a determined time.

#### 4.2.5. Degradation Test

The degradation rate of the hydrogels was analyzed by incubation in a PBS solution for 14 days. The hydrogels’ volume and the incubation conditions were the same as in the swelling test. The hydrogels were removed from the PBS solution once a day for 14 days. The excess surface water was removed using filter paper, and the weight was recorded. The completely swollen hydrogels’ weight, after incubating for 1 day, was chosen as the initial weight. The degradation rate (DR) was calculated according to the following equation

(2) $$\mathrm{DR}=\frac{Wd}{Wi}\times 100$$

where *W_i_* is the weight of initial hydrogel that was completely swollen after incubation for 1 day and *W_d_* is the weight of the degraded hydrogel after incubation at a determined time.

#### 4.2.6. Compressive Test

A hydrogel’s stiffness refers to its resistance to deformation under applied force, reflecting the rigidity or elasticity of the hydrogel’s structure. In this study, the stiffness of alginate hydrogels was measured using a compressive strength analysis [20]. The hydrogels’ rigidity was analyzed by compressing the hydrogel disk until it deformed. The compressive test was performed using the Instron E3000LT (Instron Inc., Buckinghamshire, UK) at room temperature, and the compression rate was 10 mm/min. The hydrogel samples were prepared as disks with a diameter and length of 1 cm. Compression of the hydrogel progressed until a sudden drop in the compressive stress curve appeared, implying that the hydrogel was deformed, and the value of maximal compressive stress was recorded.

### 4.3. HPLC Analysis

Aripiprazole was analyzed using an HPLC system (Waters Corp., Milford, MA, USA) consisting of a pump (PU-280), a UV-VIS spectrophotometric detector (UV-996) operating at 205 nm, an autosampler (AS-950-10) and a reverse-phase column (Hypersil Gold C18 250 × 4.60 mm). The mobile phase comprised 10 mM ammonium acetate in a deionized water–acetonitrile solution (10:90% *v*/*v*) and pumped at 1.0 mL/min. The injection volume was 20 µL, the column’s temperature was 25 °C, and the analytical time was 10 min. Aripiprazole peak was detected at 5.3 min. Each sample solution was filtered through a 0.45 µm membrane filter (Millipore Corp., Bedford, MA, USA).

### 4.4. In Vitro Dissolution Test

#### 4.4.1. Dissolution Test Using USP Apparatus II

The official USP Apparatus II was used to determine the dissolution rate for each formulation compared with a marketed product, Abilify Maintena^®^. The dissolution method was performed according to the Abilify Maintena^®^ NDA reference guidelines [40]. The dissolution medium was 900 mL of a PBS solution (pH 7.4 ± 0.1), with 0.25% SLS at 37 ± 0.5 °C. The ARP powder, Abilify Maintena^®^ and hydrogels were carefully placed in the dissolution medium. The paddle’s rotation rate was set to 50 rpm, and 3 mL of the solution was withdrawn at 0.25, 1, 2, 4, 8 and 24 h for HPLC analysis.

#### 4.4.2. Dissolution Test Using the Shaking Bath Method

The in vitro dissolution of hydrogels was also performed by incubation in a PBS solution for 14 days using a shaking bath (BS-06, JEIO TECH, Daejeon, Republic of Korea). The dissolution test conditions referred to the USP dissolution method of risperidone and goserelin implant formulations [40]. The hydrogels were placed in 50 mL of a PBS solution (pH 7.4 ± 0.1) with 0.25% sodium lauryl sulfate (SLS). ARP powder was used as control in this experiment. The incubation conditions were set to 37 ± 0.5 °C, with a rotation speed of 50 rpm. At predetermined time points, 1 mL of the solution was taken from the conical tube for HPLC analysis, and 1 mL of fresh PBS solution was replaced to maintain a constant volume during the drug release test for 14 days.

### 4.5. Injectability, Stability and In Situ Depot-Forming Behavior of Alginate Hydrogels

The optimized formulation F8 was evaluated for injectability, stability during storage and in situ depot-forming behavior.

#### 4.5.1. Injectability of Alginate Hydrogels in the Sol State

Injectability is a term related to the ease of parenteral administration of a dosing solution, involving the pressure or force required for injection, the evenness of the flow and no blockage of the syringe needle [43]. Herein, a formulation was considered “injectable” if it could easily pass through the needle without exerting considerable effort [20]. The sol state of the F8 formulation, before gelation occurs, must permit injection through a syringe needle without applying strong force. After mixing the GDL solution with the pregel solution, each formulation was filled into a 1 mL syringe and tested for injectability using a 23 G needle (Korea Vaccine Co., Ltd., Seoul, Republic of Korea).

#### 4.5.2. Viscosity of the Pregel Solution During Storage 

The stability of Formulation F8 was assessed by measuring the viscosity of the pregel solution after storing the sample at 25 °C for 1 month. The viscosity (Pa·s) of the sol state was analyzed at different shear rates (0.1–100 rad/s) at 25 °C by using the ARES-G2 rheometer (TA Instruments, New Castle, DE, USA).

#### 4.5.3. In Situ Depot-Forming Behavior of Alginate Hydrogels in Pig Neck Muscle

Pig neck muscles were used to confirm the in situ depot-formation behavior of hydrogels. Pig neck muscles were heated up to 37 °C using a water bath (BS-06, JEIO TECH, Daejeon, Republic of Korea). Formulation F8 (500 µL) was injected by intramuscular injection using a 23G syringe needle. Thirty minutes after injection, the muscle sections were cut, and images of the muscle’s surface and cross-section were obtained using a digital camera.

### 4.6. Statistical Analysis

Statistical analyses were conducted using Student’s *t*-test using SigmaPlot version 14.0 (Systat software, San Jose, CA, USA). Differences among samples were considered significant at a *p*-value < 0.05. The data are presented as the mean ± standard deviation.

## Figures and Tables

**Table 1:** Composition (g) of alginate hydrogel formulations.

Formulation Code	ARP	CaCO_3_	Alginate	PLP	GDL	PLP/GDL Ratio (*w*/*w*)	Total Water (mL)
F1	0.84	0.6	0.6	-	0.14	-	30
F2				0.26	0.14	13:7	
F3				0.28	0.14	14:7	
F4				0.3	0.14	15:7	
F5				-	0.18	-	
F6				0.26	0.18	13:9	
F7				0.28	0.18	14:9	
F8				0.3	0.18	15:9	

**Table 2:** Maximal compressive stress of alginate hydrogel formulations. Data are expressed as the mean ± standard deviation (*n* = 3).

Formulation Code	Maximal Compressive Stress (kPa)
F1	48.57 ± 3.280
F3	4.72 ± 0.145
F7	4.64 ± 0.020
F8	3.55 ± 0.060

## Data Availability

The original data presented in the study are included within the article, and further inquiries can be directed to the corresponding authors.

## Supplementary Materials

The following supporting information can be downloaded at: https://www.mdpi.com/article/10.3390/gels10120781/s1, Table S1: Statistical analysis of gelation time using Student’s *t*-test; Table S2: Statistical analysis of swelling ratio at 24 h using Student’s *t*-test; Table S3: Statistical analysis of degradation ratio at day-6 and day-14 using Student’s *t*-test.
