# Supplementary material for: Physicochemical Properties, Drug Release and In Situ Depot-Forming Behaviors of Alginate Hydrogel Containing Poorly Water-Soluble Aripiprazole
Source: Gels. 2024 Nov 29;10(12):781. doi: 10.3390/gels10120781 (PMC11675168; doi:10.3390/gels10120781)
Supplement: Supplementary file 1 [file gels-10-00781-s001.zip › gels-3332920-supplementary.pdf]

Table S1. Statistical analysis of gelation time using Student's t-test

Comparison	p-value	95% two-tailed confidence interval	Sample Size
F1 vs F2	0.155	(-0.259, 0.059)	3, 3
F1 vs F3	0.0093	(-4.751, -1.859)	3, 3
F1 vs F4	0.0051	(-26.428, -13.904)	3, 3
F1 vs F5	0.0135	(0.165, 0.503)	3, 3
F1 vs F6	0.0135	(0.165, 0.503)	3, 3
F1 vs F7	0.0067	(-1.652, -0.570)	3, 3
F1 vs F8	0.0049	(-11.186, -5.924)	3, 3

Table S2. Statistical analysis of swelling ratio at 24h using Student's t-test

Comparison	p-value	95% two-tailed confidence interval	Sample Size
F1 vs F3	0.382	(-1.362, 0.808)	3, 3
F1 vs F7	0.696	(-0.811, 0.659)	3, 3
F1 vs F8	0.671	(-0.479, 0.599)	3, 3
F3 vs F7	0.544	(-0.681, 1.083)	3, 3
F3 vs F8	0.313	(-0.850, 1.524)	3, 3
F7 vs F8	0.545	(-0.455, 0.727)	3, 3

Table S3. Statistical analysis of degradation ratio at day-6 and day-14 using Student's t-test

	Comparison	p-value	95% two-tailed confidence interval	Sample Size
Day-6	F1 vs F3	0.519	(-17.622, 28.440)	3, 3
	F1 vs F7	0.215	(-28.510, 63.738)	3, 3
	F1 vs F8	0.123	(-4.149, 23.471)	3, 3
Day-14	F1 vs F3	0.423	(-7.673, 4.779)	3, 3
	F1 vs F7	0.423	(-15.584, 9.706)	3, 3
	F1 vs F8	0.0015	(-8.295, -5.937)	3, 3
